# Epidemiological features and spatial clusters of hand, foot, and mouth disease in Qinghai Province, China, 2009–2015

**DOI:** 10.1186/s12879-018-3509-7

**Published:** 2018-12-05

**Authors:** Lili Xu, Yan Shi, Jeanette J. Rainey, Zhijie Zhang, Huayi Zhang, Jinhua Zhao, Yonghong Li, Huaxiang Rao, Yanming Li, Qiaohong Liao, Yongcheng Ma

**Affiliations:** 1 Institute for Infectious Disease Control and Prevention, Qinghai Provincial Center for Disease Control and Prevention, Xining, Qinghai China; 2 Department of Epidemiology and Biostatistics, School of Public Health, Fudan University, Shanghai, China; 3 International Emerging Infections Program, Division of Global Health Protection, United States Centers for Disease Control and Prevention, Beijing, China; 4 Division of Infectious Disease, Key Laboratory of Surveillance and Early Warning on Infectious Disease, Chinese Center for Disease Control and Prevention, Beijing, China; 5 Division of Global Health Protection, Center for Global Health, Centers for Disease Control and Prevention, Atlanta, USA

**Keywords:** HFMD, Cyclical pattern, Spatial clusters, Qinghai Province

## Abstract

**Background:**

Hand, Foot, and Mouth Disease (HFMD) is most frequently caused by Enterovirus71 (EV-A71) or Coxsackie virus A16 (CV-A16), infants and young children are at greatest risk. Describing the epidemiology of HFMD can help develop and better target interventions, including the use of pediatric EV-A71 vaccination.

**Methods:**

We obtained data from the national surveillance system for HFMD cases with onset dates from 2009 to 2015. We defined probable cases as patient with skin papular or vesicular rashes on the hands, feet, mouth, or buttocks and confirmed cases as patients with the above symptoms along with laboratory-based enterovirus detection. We generated overall and age-specific annual incidence rates and described the temporal variability and seasonality of HFMD in Qinghai Province. We identified spatial clustering of HFMD incidence at the county level using the Local Indicator of Spatial Associationand an alpha level of 0.05.

**Results:**

During the study period, 14,480 HFMD probable or confirmed cases were reported in Qinghai Province. Of the 2158 (14.9%) with laboratory confirmation, 924 (42.6%) were caused by CV-A16 and 830 (38.2%) were caused by EV-A71. The majority (89%) of all case-patients were ≤ 5 years of age and male (61.5%). The overall mean annual HFMD incidence rate was 36.4 cases per 100,000 populations, while the incidence rate for children ≤5 years of age was 379.5 cases per 100,000. Case reports peaked during the months of May through July. HFMD was predominantly caused by EV-A71, except in 2010 and 2014 when CV-A16 was the predominant causative agent. High incidence rates of HFMD were clustered (Moran’s *I* = 0.59, *P <* 0.05) in the eastern region of the province.

**Conclusion:**

HFMD remains an important cause of childhood disease in Qinghai Province, occurring in an acyclical pattern of increased incidence, primarily due to CV-A16 circulation every three years. Incidence is also seasonal and tends to spatially cluster in the eastern region of the province. Since approximately 40% of confirmed HFMD cases were due to EV-A71, EV-A71 vaccination is likely to have a positive impact on the HFMD disease burden. Routine analysis of local surveillance data is crucial for describing disease occurrence and changes in etiology.

**Electronic supplementary material:**

The online version of this article (10.1186/s12879-018-3509-7) contains supplementary material, which is available to authorized users.

## Background

Hand, foot, and mouth disease (HFMD) is a common infection primarily caused by Enterovirus71 (EV-A71), Coxsackie virus A16 (CV-A16), and, to a lesser extent, CV-A6. Clinical manifestations typically include fever, skin eruptions on hands and feet, and vesicles in the mouth; approximately 30–90% of infections may be asymptomatic [1–5]. Approximately two million cases of HFMD are reported in China each year [6]. Most infections are mild and self-limiting. However, infections with EV-A17 can cause neurological and systemic complications as well as death [7–9]. Because enterovirus transmission occurs through direct contact with saliva, feces, vesicular fluid or respiratory droplets of an infected individual, or through indirect contact via contaminated articles [10], young children are particularly at risk. Outbreaks are frequently linked to nursery and pre-school attendance [11].

HFMD outbreaks have occurred across the Asia-Pacific region, including China, since 1997 [12–16]. Large outbreaks in Shandong and Anhui Provinces in 2007 and 2008 were associated with thousands of cases and resulted in 36 deaths in infants and young children [17, 18]. Because of these outbreaks, China established a case-based national surveillance system for HFMD in May 2008. Health care workers at medical facilities at the local, prefecture, and provincial levels are required to report cases within 24 h of diagnosis. Additionally, three inactivated monovalent EV-A71 vaccines were licensed in China in 2016 for children aged 6–59 months [19]. In 2017, health officials in Qinghai introduced the vaccine as category 2 vaccine (i.e. voluntary vaccination covered as an out-of-pocket expense).

Better descriptions of the epidemiology of HFMD, particularly at the provincial level, can help identify and better target appropriate interventions, including the potential benefits of pediatric EV-A71 vaccination on reducing unnecessary moribidty and associated economic costs [20, 21]. This paper describes the analysis of HFMD surveillance data from Qinghai Province; estimates the temporal and spatial distribution of reported HFMD cases,and examines the etiology and age distribution from available case reports. Findings from this analysis can serve as a baseline assessment of HFMD for estimating the impact of the vaccine introduced in 2017, and guide efforts for targeting populations at highest risk for disease.

## Methods

### Study area

**Fig. 1:**
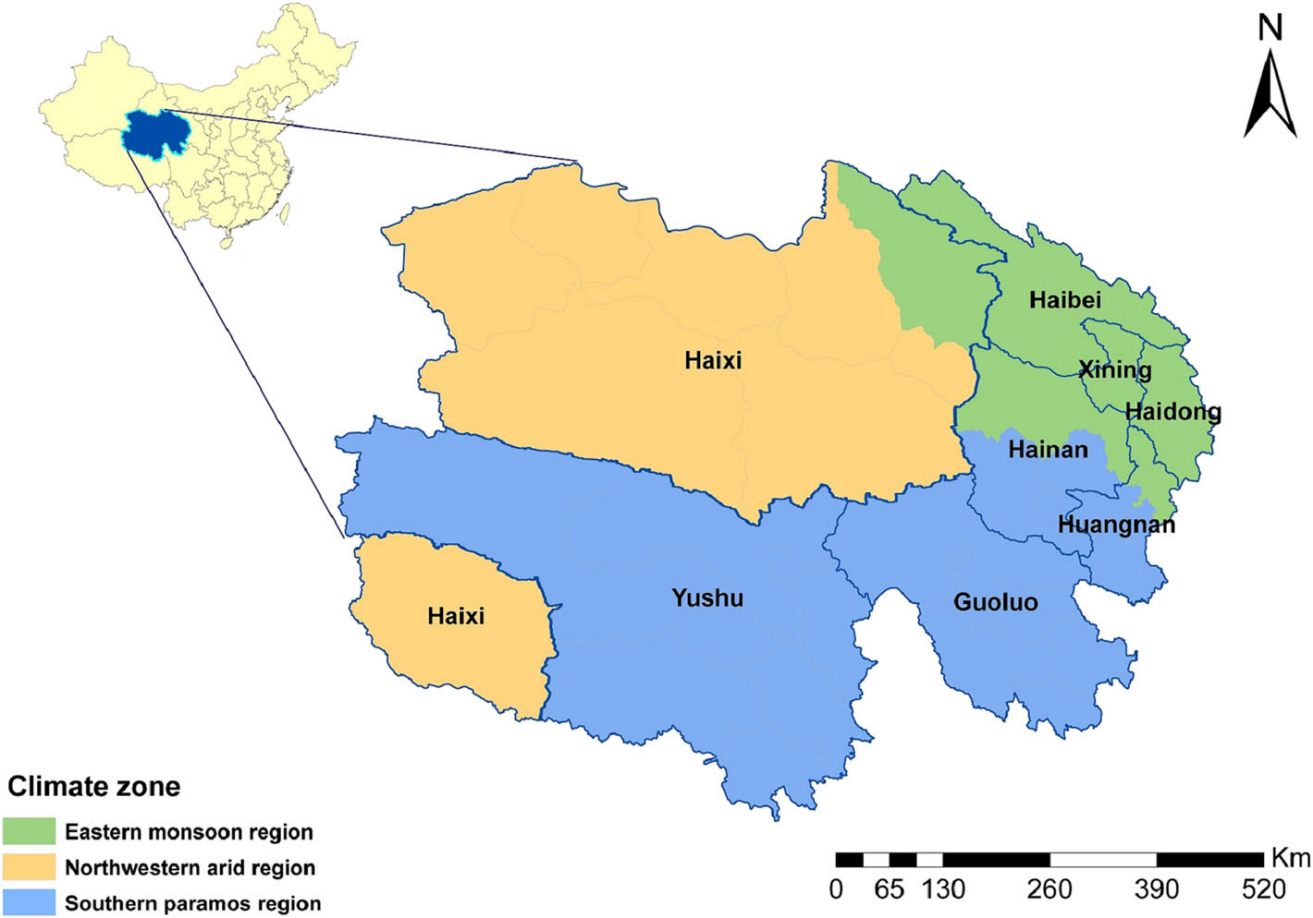
Map of Qinghai Province, China by climate zone and prefecture

Qinghai Province is located in the northeastern Tibetan Plateau, and is administratively divided into eight prefectures and 46 counties. In 2015, the population was 5.8 million with approximately 440,000 (7.6%) children 0–5 years of age. Nearly 70% of the population lives in the eastern region of the province around Xining City (the capital of Qinghai) and Haidong City. The provincial population density is approximately 8.1 residents (range from 0.4 to 11,558) per square kilometer. Qinghai Province can be divided into three regions according to the geographical location and climate characteristics: the eastern monsoon region, the northwestern arid region, and the southern paramos (high and cold) region [22] (Fig. 1). Children aged 3–6 years frequently attend pre-school or kindergarten [23]. A non-working parent or grandparent often provides childcare for children less than 3 years old.

### Data collection

Qinghai HFMD case-based surveillance data for 2009 through 2015 were obtained from the Chinese Information System for Disease Control and Prevention (CISDCP). Surveillance data include information on case-patient demographics (birth date, gender, address), symptom onset date, diagnosis date, hospitalization date, disease severity, and clinical outcome.

We collected information on the etiology of a subset of reported HFMD cases. Hospital staff in all 46 counties were required (per HFMD surveillance guidelines) to test clinical samples from the first five patients each month presenting with mild, probable disease [24]. Hospital staff also attempted to obtain and test specimens from all severe cases and HFMD cases resulting in death. In 2011, sentinel surveillance was established in two hospitals in Xining and one hospital in Haidong City. Samples from at least 10 HFMD cases were collected and tested from each hospital every week for the pathogen detection and laboratory confirmation.

All specimens collected from the sentinel hospitals and counties from 2009 to 2010 were sent to the provincial-level Center for Disease Control and Prevention (CDC) for testing. Specimens collected by county-level hospitals were sent to the corresponding prefecture CDCs after 2011. EV-A71 and CV-A16 were detected by Reverse Transcription Polymerase Chain Reaction (RT-PCR) according to standard protocols disseminated by China CDC [25]. After 2013, pan-enteroviruses, EV-A71, CV-A16 were tested simultaneously in all laboratories. All positive specimens detected by the Prefecture CDCs were sent to the provincial laboratory for verification and virus isolation.

We obtained annual county-level population data from the Qinghai Provincial Bureau of Statistics for calculating incidence rates. Geographical boundary files were obtained from the National Fundamental Geographic Information System of China.

### Data management and analysis

We linked available laboratory results to case-based data obtained from the national HFMD surveillance system for Qinghai Province by name, date of birth, and date of diagnosis. All probable and confirmed case-patients with illness onset dates from January 1, 2009 to December 31, 2015 were included in the analysis. A probable case was defined as a patient with skin papular or vesicular rashes on the hands, feet, mouth, or buttocks, with or without fever. A confirmed case was defined as a probable case with laboratory evidence of enterovirus infection (including isolation of enterovirus or detection of enterovirus-specific RNA). Probable and confirmed cases were further subdivided into severe and mild cases. Case-patients presenting with neurological complications (e.g., aseptic meningitis, encephalitis, encephalomyelitis, or acute flaccid paralysis), and/or cardiopulmonary complications (pulmonary oedema, pulmonary haemorrhage, or cardiorespiratory failure) were classified as severe; otherwise, case-patients were classified as mild [26].

We analyzed the age and gender distribution of reported cases and the etiology of laboratory confirmed cases. To quantify seasonal patterns by prefecture, we created heat maps for reported cases for each week of the year using a range (maximum and minimum) normalization method. All data were converted into an N (0,1) distribution in order to better compare variability of HFMD case-reports over time in the heat maps. We calculated annual incidence rates for the total population and for children 0–5 years of age using the total number of cases reported each year divided by the total and age-specific county, regional, and provincial-level population estimates. HFMD incidence rates were mapped using ArcGIS 10.0 (ESRI Inc., Redlands, CA, USA). We used the software R 3.2.3 (https://www.r-project.org/) to perform the statistical analyses and used an alpha level of 0.05 to define statistical significance.

We used GeoDa spatial analytic software (http://geodacenter.github.io/download.html, GeoDa Center for Geospatial Analysis and Computation, Arizona State University, AZ, USA) to identify significant spatial clusters of HFMD. Local Indicators of Spatial Association (LISA) [27] were generated to determine the location of spatial clusters of counties with similar HFMD incidence rates for children less than 6 years of age. The null hypothesis was that there was no association in HFMD rates between neighboring counties. We applied an alpha level of 0.05 following 999 random permutations to test the statistical significance of identified clusters. Spatial weights were created using the queen contiguity rule.

## Results

### HFMD case characteristics and etiology

Qinghai health officials reported 14,480 HFMD cases in Qinghai between January 1, 2009 and December 31, 2015. Of these cases, 12,859 (88.8%) were in children 0–5 years of age and 61.5% were male (Table 1). Seven cases were defined as severe and three of those were fatal. The fatal cases occurred in children 9 months, 2 years, and 4 years of age.

**Table 1:** Demographic characteristics and etiology of Hand, Foot, and Mouth Disease, Qinghai Province, 2009–2015 (*n* = 14,480)

	2009 *n* (%)	2010 *n* (%)	2011 *n* (%)	2012 *n* (%)	2013 *n* (%)	2014 *n* (%)	2015 *n* (%)	Total *n* (%)
Age group								
<6 month	13 (0.5)	30 (0.7)	4 (0.7)	9 (1.1)	17 (1.0)	26 (1.0)	11 (0.6)	110 (0.8)
6–11 month	120 (4.9)	564 (12.4)	38 (6.3)	36 (4.5)	247 (14.7)	172 (6.4)	163 (9.4)	1340 (9.3)
1–3 years	1468 (60.3)	2449 (53.7)	354 (58.7)	496 (62.6)	1004 (59.7)	1457 (54.4)	1009 (58.4)	8237 (56.9)
4–5 years	602 (24.7)	1040 (22.8)	152 (25.2)	174 (22.0)	241 (14.3)	651 (24.3)	312 (18.1)	3172 (21.9)
6–9 years	184 (7.6)	352 (7.7)	40 (6.6)	51 (6.4)	127 (7.6)	281 (10.5)	148 (8.6)	1183 (8.2)
10– 14 years	39 (1.6)	98 (2.1)	10 (1.7)	20 (2.5)	40 (2.4)	73 (2.7)	66 (3.8)	346 (2.4)
≥15 years	10 (0.4)	28 (0.6)	5 (0.8)	6 (0.8)	5 (0.3)	20 (0.7)	18 (1.0)	92 (0.6)
Gender								
Male	1542 (63.3)	2828 (62.0)	371 (61.5)	495 (62.5)	1002 (59.6)	1612 (60.1)	1056 (61.1)	8906 (61.5)
Female	894 (36.7)	1733 (38.0)	232 (38.5)	297 (37.5)	679 (40.4)	1068 (39.9)	671 (38.9)	5574 (38.5)
Gender ratio	1.72:1	1.63:1	1.60:1	1.67:1	1.48:1	1.51:1	1.57:1	1.60:1
Case definition								
Probable cases	2339 (96.0)	4469 (98.0)	568 (94.2)	633 (79.9)	1329 (79.1)	1851 (69.1)	1121 (64.9)	1,2310 (85.0)
Confirmed cases^b^	97 (4.0)	92 (2.0)	35 (5.8)	159 (20.1)	352 (20.9)	829 (30.9)	606 (35.1)	2170 (15.0)
Etiology^a^								
EV-A71	56 (57.7)	18 (19.6)	27 (77.1)	149 (93.7)	224 (63.6)	108 (13.0)	248 (40.9)	830 (38.2)
CV-A16	41 (42.3)	74 (80.4)	8 (22.9)	10 (6.3)	65 (18.5)	588 (70.9)	138 (22.8)	924 (42.6)
Other EV	0 (0.0)^a^	0 (0.0)^a^	0 (0.0)^a^	0 (0.0)^a^	63 (17.9)	133 (16.0)	220 (36.3)	416 (19.2)
Total	2436 (100.0)	4561 (100.0)	603 (100.0)	792 (100.0)	1681 (100.0)	2680 (100.0)	1727 (100.0)	14,480 (100.0)

^a^2009 to 2012, laboratory testing was performed only for EV-A71 and CV-A16 enteroviruses

^b^Confirmed cases are from the first 5 cases of the month in each county and the 10 cases of every week in three sentinel hospitals of the province

**Fig. 2:**
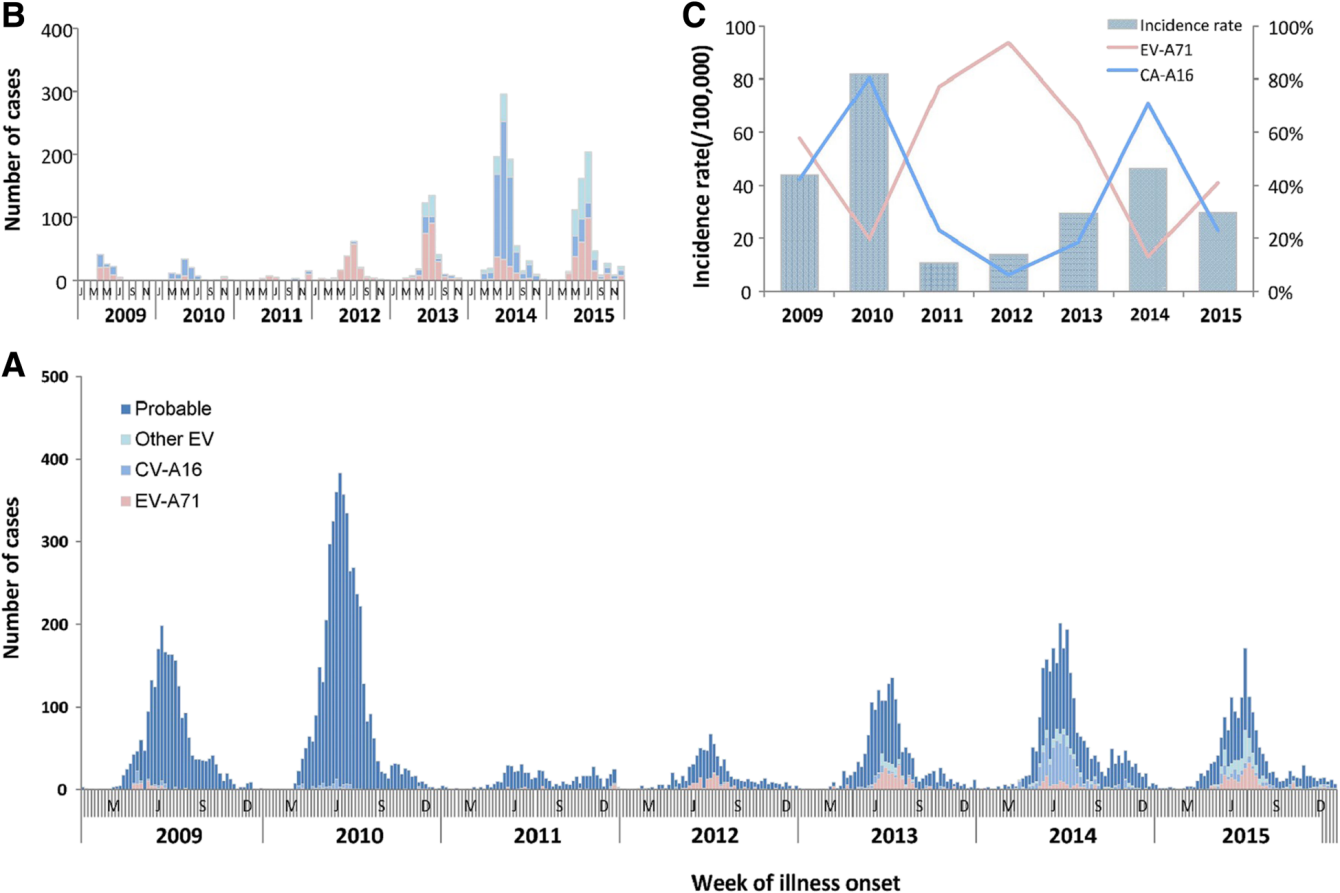
Reported cases Hand Foot and Mouth Disease, Qinghai, China, 2009–2015, by etiology and year (*n* = 14,480). (**a**) Distribution of Hand, Foot, and Mouth Disease by case definition (probable and confirmed) and etiology of laboratory-confirmed cases by year. (**b**) Distribution of Hand, Foot, and Mouth Disease of laboratory-confirmed cases by year. (**c**) Distribution of CV-A16 and EV-A71 etiology among laboratory-confirmed cases and overall incidence rate of HFMD by year

Specimens were collected from 3715 (25.7%) of the 14,480 reported cases. Of these, 2158 (14.9%) were positive for an enterovirus infection, of which, 924 (42.6%) were positive for CV-A16 and 830 (38.2%) were positive for EV-A71 (including 6 positive for both CV-A16 and EV-A71). The remaining 416 (19.2%) were positive for other enteroviruses. Of the seven severe cases, five (including the three deaths) were due to EV-A71, and two were lacking specimens.The annual variability of HFMD by etiology is presented in Fig. 2 and Additional file 1: Figure. HFMD was predominantly caused by EV-A71, except in 2010 and 2014 when CV-A16 was the predominant causative agent, representing 80.4 and 70.9% of reported cases in each year, respectively. During 2013 to 2015, the percentage of other enteroviruses increased from 17.9 to 36.3%.

### Incidence rates, secular trends and seasonality

**Fig. 3:**
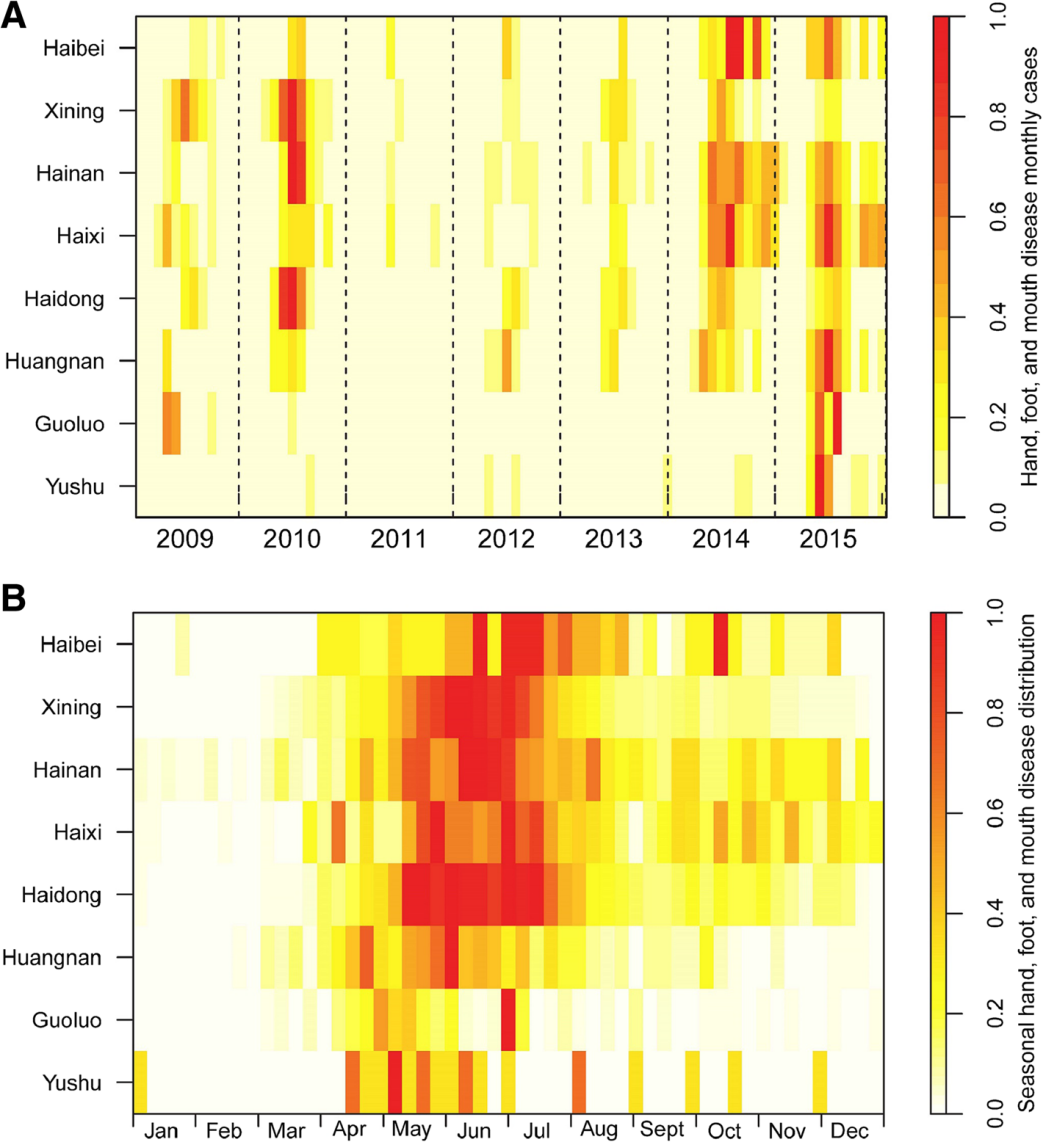
Heatmap of reported cases of Hand, Foot, and Mouth Disease by prefecture and year, Qinghai Province, China, 2009–2015 (n = 14,480). (**a**) Time series of monthly reported cases of HFMD, standardized by the number of annual cases. (**b**) Seasonal distribution of cases of HFMD, standardized by range (maximum and minimum) normalization method from 2009 to 2015. For (A) and (**b**), prefectures were ordered top to bottom by latitude from north to south

The mean seven-year annual incidence rate of HFMD in Qinghai was 36.4 cases per 100,000 population and 379.5 cases per 100,000 children. The incidence rates ranged from a low of 121.6 cases per 100,000 children in 2011 to 885.6 per 100,000 population in 2010 (Table 2, Fig. 2, and Additional file 2: Table). For all age-groups, HFMD case-reports generally peaked between the months of May to July. Cases during this three-month period accounted for 58.3% of the total number of reported cases in 2012 and 71.0% in 2010. Peak incidence occurred 3.2 weeks earlier (range: 1–6 weeks) in the southern prefectures than in the northern prefectures of province (Fig. 3).

**Table 2:** Annual incidence rates,severity, and case-fatalityof Hand, Foot, and Mouth Disease (HFMD), Qinghai Province, 2009–2015

Year	Number of Cases	Number of Severe Cases	Number of Deaths	Incidence Rate(/100,000)	Percent of cases with severe disease(%)	Case-fatality(%)^a^
2009	2436	0	0	44.0	0.00	0.00
2010	4561	1	0	81.8	0.02	0.00
2011	603	0	0	10.7	0.00	0.00
2012	792	0	0	13.9	0.00	0.00
2013	1681	1	1	29.4	0.06	0.06
2014	2680	3	1	46.4	0.11	0.04
2015	1727	2	1	29.6	0.12	0.06
Total	14,480	7	3	36.4	0.05	0.02

^a^Case-fatality calculated as the number of deaths divided the total number of cases reported each year

### Geographic distribution of HFMD incidence and spatial clustering

**Fig. 4:**
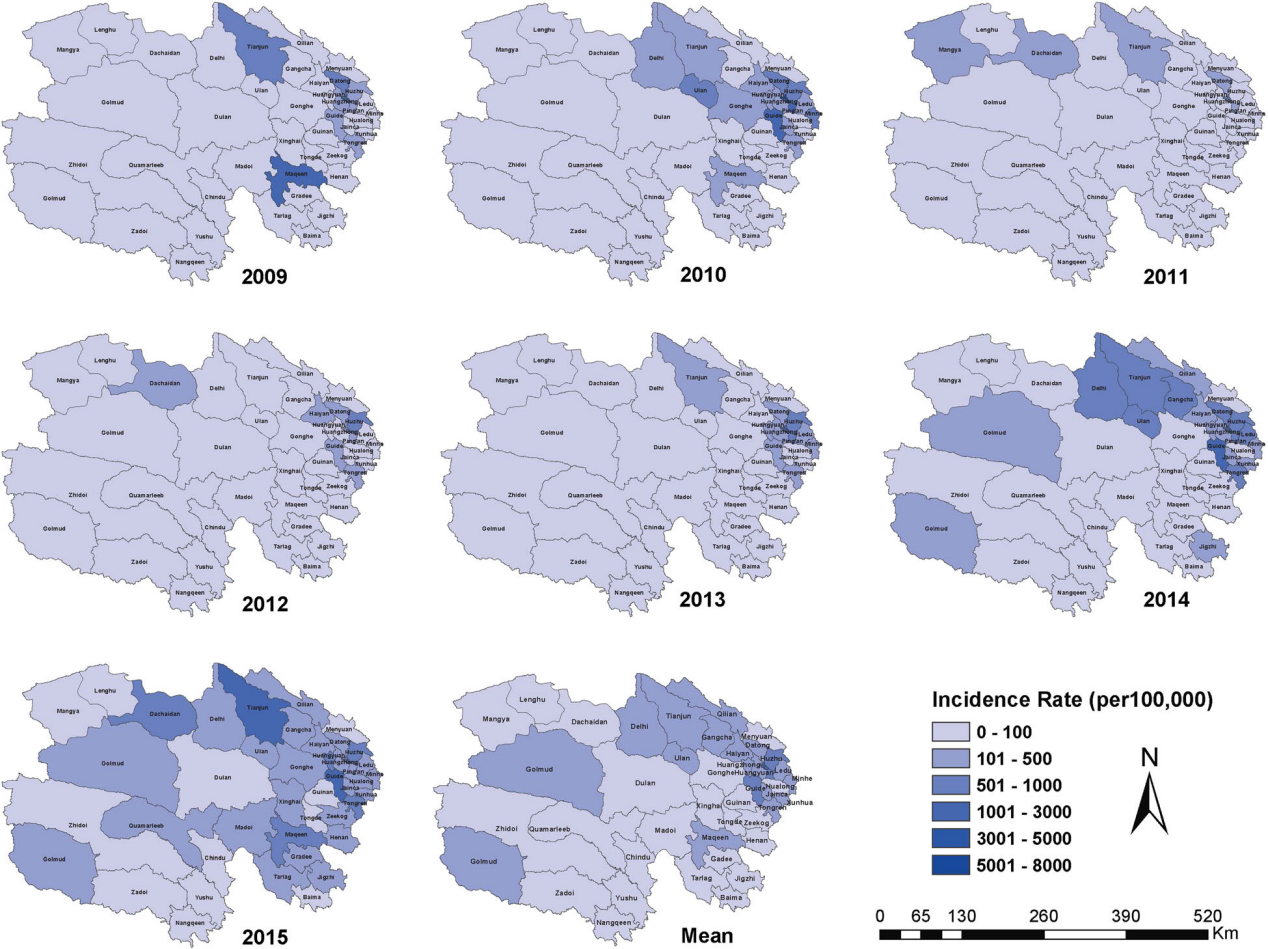
Annual and mean seven-year county-level incidence rates of HFMD for children 0 to 5 years of age, Qinghai Province, China, 2009–2015

The geographic distribution of HFMD in all age-groups and children 0–5 years of age varied substantially in Qinghai Province (Fig. 4 and Additional file 3: Figure). Overall, incidence rates were highest in densely populated counties in the the eastern regions (which, includes Xining and Haidong Cities). The mean annual incidence rates for the region was 625.1 with county rates ranging from 227.6 to 1113.3 cases per 100,000 children. Counties located in the western and southern regions had lower mean incidence rates, ranging from 29.3 to 362.5 cases and 3.1 to 131.2 cases per 100,000, respectively. During 2011 and 2012, no cases were reported from 12 of the 46 of mostly southern (high and cold) Yushu and Guoluo Prefectures.

**Fig. 5:**
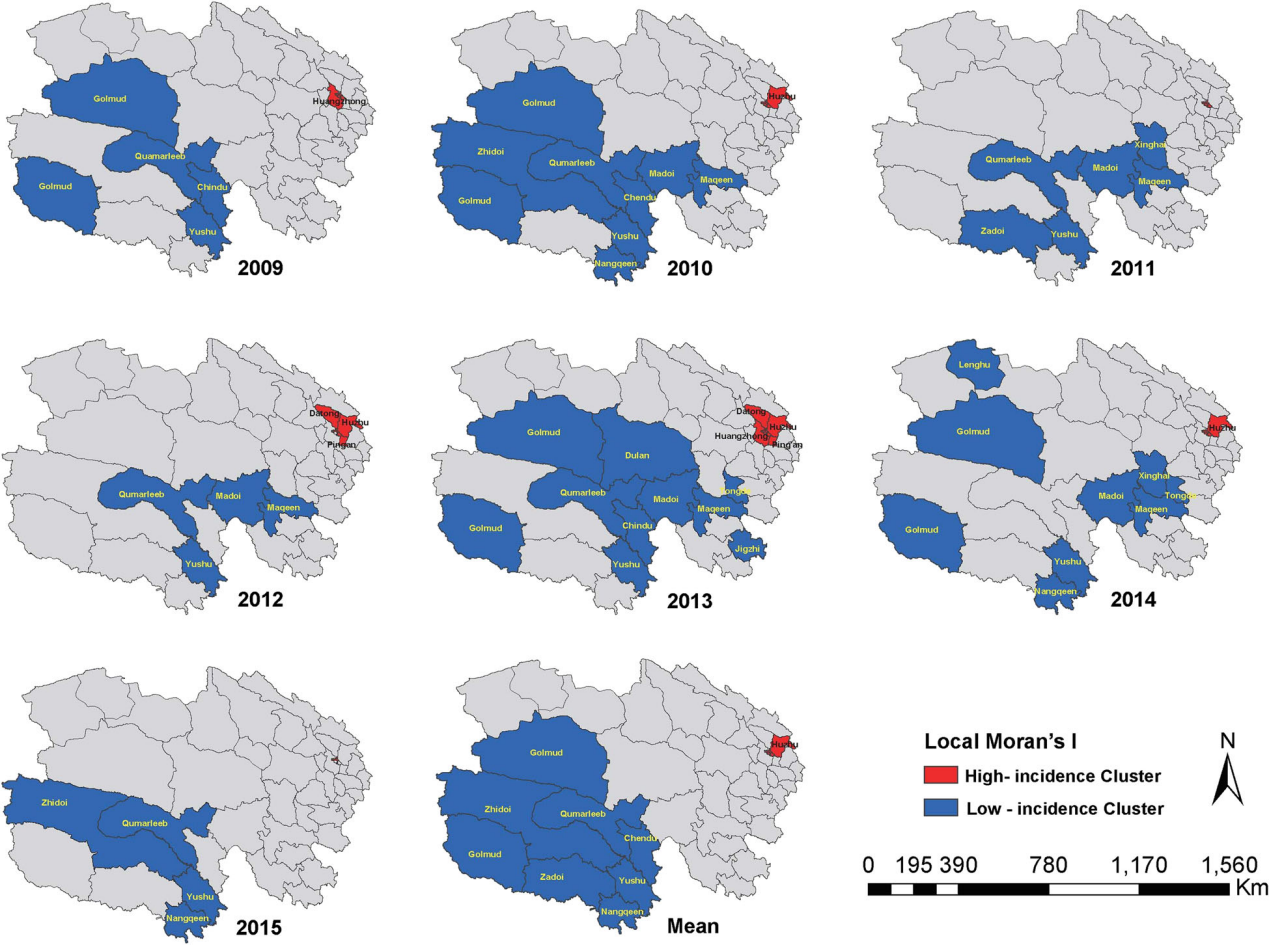
Statistically significant county-level high incidence and low incidence spatial clusters of Hand, Food, and Mouth Disease, for children 0 to 5 years of age, Qinghai Province, 2009–2015. Clusters detected using the Local Indicator for Spatial Autocorrelation (LISA) in GeoDa statistical software (http://geodacenter.github.io/download.html)

Using the seven-year mean annual HFMD incidence rate for children 0–5 years of age, we identified five county-level clusters of high HFMD incidence and seven county-level clusters of low HFMD incidence (Table 3, Fig. 5). All of the high-incidence clusters were located in the densely populated eastern monsoon region, including four districts (Chengdong, Chengbei, Chengzhong, Chengxi – with more than 3500 persons per square km) of Xining City and one county (Huzhu– with 110 persons per square km) of Haidong City. Low incidence clusters were located in the southern paramos region (including six counties of Yushu Prefecture – with approximately 2 persons per square km) and the northwest arid region (one county of Haixi Prefecture – with 1.9 persons per square km). Similar findings were generated when HFMD incidence rates were analyzed separately for each year (see Additional file 4: Table S1-7).

**Table 3:** Statistically significant county-level high incidence and low incidence spatial clusters of Hand, Foot, and Mouth Disease identified using the Local Indicator for Spatial Autocorrelation in GeoDa software (http://geodacenter.github.io/download.html)

Type	County Name	Mean IR^a^	Local Moran	*P-*value
High-Risk Cluster	Huzhu	511.0	0.47	0.002
	Chengdong	1902.0	4.78	0.001
	Chengzhong	2040.8	7.08	0.001
	Chengbei	1849.5	4.80	0.001
	Chengxi	1387.0	4.55	0.005
Low-Risk Cluster	Chindu	0.0	0.31	0.034
	Zhidoi	12.8	0.28	0.043
	Nangqeen	12.7	0.33	0.007
	Qumarleeb	22.0	0.27	0.013
	Yushu	4.3	0.33	0.001
	Golmud	155.1	0.15	0.003
	Zadoi	3.9	0.29	0.043

^a^*IR* Incidence Rate, per 100,000 children 0–5 years of age

Rates were calculated as the mean annual incidence rate for children less than 6 years of age for the seven-year study period

## Discussion

Our epidemiologic and spatial-temporal analysis confirms that HFMD, with mean annual incidence rates of 379.5 cases per 100,000 in children 0 to 5 years of age, remains an important public health issue for children in Qinghai Province. High incidence counties were all located in the eastern region of the province. Although less than half of laboratory confirmed cases were due to EV-A71, EV-A71 vaccine is still likely to have a positive impact on reducing the occurrence of HFMD, including decreasing unnecessary economic costs and use of health care servces resulting from mild or moderate HFMD cases [20, 21]. Annual changes in the predominant causative agent are noteworthy, including recent increases in non-EVA71 and non-CV-A16 enteroviruses.

Findings from our spatial analysis are consistent with findings from studies conducted in other provinces in China, such as in Guangdong [28], Sichuan [29], and Guangxi [30]. All of these provincial-level analyses detected high-risk clusters primarily in provincial capitals and surrounding counties and districts [28–31]. Similar to the high-risk clusters detected in and around Xining and Haidong City, Qinghai Province, in Guangdong and Sichuan, for example, high-risk clusters were identified in and around Guangzhou and the Pear River Delta [28] and Chengdu [29], respectively. Case clustering in these provincial centres suggests the role of human mobility and high population densities in HFMD transmission. These factors, along with general industrialization, which was also identified with case-clustering in Liuzhou City, Guangxi [30], are linked to high levels of social mixing, increasing the chance of introduction of an infectious case to the population as well as of contact between an infectious case and the susceptible population [32]. Xining and Haidong Cities, similar to Guangzhou in Guangdong Province, Chengdu in Sichuan Province, and Nanning, Guangxi Province are major travel and trading centres and have population densities of at least 3500 and 110 persons per square kilometer, respectively. Similar findings were observed in a spatial-temporal anlaysis of HFMD at the national level [31].

Despite similarities in case-clustering, the overall mean annual incidence rates in Guangdong (175.1 per 100,000 population), Zhejiang (173.0), Guangxi (298.3), and Sichuan (43.65) were substantially higher than in Qinghai (36.4). Although the overall incidence rates differ, in each province between approximately 87 and 95% of reported HFMD cases were in children 0–5 years of age [26–29]. This is similar to Qinghai where 88% of reported cases were children 0–5 years of age. Differences in incidence rates are likely to reflect the age-distribution of the population and social mixing patterns in both urban and rural regions of these provinces [28–30, 33]. Differences could also reflect health seeking behavior and surveillance and HFMD reporting practices and possibly climate related factors. In all locations, the incidence rates were the highest in children 0 to 5 years of age.

We detected a single spring-summer peak between May to July in HFMD case-reporting in Qinghai. This is similar to patterns seen elsewhere in northern China, including Shandong Province [34] and to a lesser degree, in Guangxi Province [30] as well as elsewhere in temperate Asia including Japan [24, 35–38]. In these other locations, the seasonal pattern of HFMD has been statistically linked to increasing humidity, lack of sunshine, and rainfall [24]. Although we do not include specific meteorological data in our analysis, our findings are consistent with increasing higher relative humidity in the eastern monsoon region in late spring and early summer and approximately one month later in other locations in the province. The two-peak HFMD season in summer and winter in Guangdong Province occurred during the school holidays when children have less contact with other children [28]. HFMD transmission could be linked to both virus properties (i.e., preference for humidity) [24, 28, 36, 39–41] and social mixing [42, 43]. Future research in Qinghai should focus on collecting meteorological and contact survey data in order to further evaluate these hypotheses.

EV-A71 was the predominant cause of HFMD in Qinghai each year during the study period, with the exception of 2010 and 2014. During these two years, the majority of cases were due to CV-A16. This differs from the yearly distribution of CV-A16 in Guangdong (predominant in 2009) [29], Guangxi (2011) [30] as well as in Shandong (2010) Provinces [34]. However, the cyclical nature of enteroviruses and Coxsackie viruses in all locations could be due to duration of protective immunity following infection (including duration of cross-protection of EV-A71 and CV-A16) as well as variability in serotypes and accumulation of susceptible children [44, 45]. At the same time, recent increases in non-EVA71 and non-CV-A16 enteroviruses in Qinghai as well as elsewhere in China [46, 47] suggest local serotype replacement may occur rapidly, highlighting the importance of provincial and county level laboratory-based surveillance.

According to a serological survey in Xining City in 2009, the positive rate and geometric mean titers of EV-A71 neutralizing antibody were lowest in the children aged less than 1 year and gradually increased with age [48]. Approximately 70% of children had serologic evidence of past EV-A71 infection by 5 years of age [49]. These findings are consistent with the age distribution of cases in Qinghai, where approximately 88% of cases were less than 6 years of age. The male to female case gender ratio in Qinghai was 1.60:1, similar to the gender ratio observed in other provincial-level studies [29, 33, 41].

For our spatial analysis, we generated Local Indicators of Spatial Association (LISA) to identify spatial autocorrelation - county-level clusters - of high HFMD incidence rates among children 0–5 years of age. The LISA test for spatial auto-correlation is less sensitive compared to the spatial scan test [28, 50] in accounting for unique geographic features such as Qinghai Lake. However, LISA tests examine clustering according to user defined neighbor relationships. For this study, we defined neighbors using the queen contiguity weights. Different neighbor relationships and weights can impact cluster detection. Additionally, we conducted our spatial analysis using age-specific incidence rates to focus on children less than 5 years of age. Both of these factors differentiate this study from a previously conducted study that found a lack of statistically significant county-level clusters of high HFMD incidence in Qinghai [31].

Despite a number of similarities with other provinces, HFMD transmission in Qinghai is associated with distinct regional features and etiologic variability, underscoring the importance of local surveillance data. Since Qinghai health officials introduced the EV-A71 vaccine as a category-2 vaccine (cost covered by ‘out-of-pocket’) in 2017, coverage has remained low. Our provincial and regional estimates can provide baseline estimates of HFMD in Qinghai for future vaccine impact assessments, including shifts in etiologic distribution, and evaluations on equitable access [51] to the vaccine in the province. Public health officials should continue to strengthen current HFMD prevention and control strategies, particularly prior to and during the peak months of HFMD transmission.

This study has several limitations. First, we were only able to include clinical cases reported to surveillance system in this analysis. As a consequence, we assumed that the geographic distribution of asymptomatic cases, approximately 10 to 70% of all infections [2], was similar to clinical cases. Second, the quality of surveillance data may have differed by region. Assessing the level of variability in case detection and reporting was beyond the scope of this study, but should be considered in future HFMD research projects, including exploring possible regional variability in health care seeking behavior. Third, the surveillance system did not test for non-EV-A71 and non-CV-A16 enteroviruses from 2009 to 2012. The enterovirus serotype distribution of EV-A71 and CV-A16 during these years, therefore, are estimates only. Fourth, fewer specimens were collected from 2009 to 2010 compared to other years. This was primarily driven by implementation of the HFMD surveillance program and impact of the influenza A(H1N1) pandemic in 2009, and the large earthquake in Yushu in 2010. Nevertheless, specimens were collected from most counties in the province and sentinel hospitals on a monthly basis, providing a reasonably representative sample of HFMD cases during the study period. Finally, we only used one spatial-temporal cluster detection method. While using more than one method can strengthen the robustness of the results, our findings are consistent the descriptive characteristics of high-risk clusters identified from other provinces. Therefore, our results were unlikely statistically impacted by this limitation.

## Conclusion

HFMD remains an important childhood disease in Qinghai Province, occurring in an acyclical pattern of increased HFMD incidence, primarily due CV-A16 circulation every three years. Incidence is most common in May through July each year and spatially clusters in the eastern region of the Province. Since more than 40% of confirmed HFMD cases are due to EV-A71, EV-A71 vaccination is likely to have a positive impact on the HFMD disease burden. Based on our findings, prefectures and counties in the eastern region should be prioritized for additional HFMD prevention and control interventions. However, additional studies should be conducted to elucidate the role of other factors, including health care seeking behavior, social mixing patterns and contact rates of children, and the impact of climate. We strongly recommend routine analysis of local surveillance data to better identify changes in disease occurrence and underlying etiology.

## Additional files

Additional files 1: **Figure.** Proportions of enterovirus serotypes in laboratory-confirmed cases of HFMD in 2009–2015, Qinghai Province, China. (DOCX 96 kb)

Additional files 2: **Table.** Annual incidence rates, severity, and case-fatality for children 0–5 years of age of Hand, Foot, and Mouth Disease (HFMD), Qinghai Province, 2009–2015. (DOCX 13 kb)

Additional files 3: **Figure.** Annual and mean seven-year county level incidence rates of HFMD, Qinghai Province, China, 2009–2015. (DOCX 220 kb)

Additional files 4: **Tables S1–7.** Statistically significant county-level high incidence and low incidence spatial clusters of Hand, Foot, and Mouth Disease identified using the Local Indicator for Spatial Autocorrelation in GeoDa software in 2009–2015. (DOCX 24 kb)

## Abbreviations

CDC: Center for Disease Control and Prevention
CISDCP: Chinese Information System for Disease Control and Prevention
CV-A16: Coxsackie virus A16
EV-A71: Enterovirus71
HFMD: Hand, foot, and mouth disease
LISA: Local Indicators of Spatial Association
RT-PCR: Reverse Transcription Polymerase Chain Reaction

## References

[1] Kogon A, Spigland I, Frothingham TE, Elveback L, Williams C, Hall CE, Fox JP (1969). The virus watch program: a continuing surveillance of viral infections in metropolitan New York families. VII. Observations on viral excretion, seroimmunity, intrafamilial spread and illness association in coxsackie and echovirus infections. Am J Epidemiol.

[2] Chang LY, Tsao KC, Hsia SH, Shih SR, Huang CG, Chan WK, Hsu KH, Fang TY, Huang YC, Lin TY (2004). Transmission and clinical features of enterovirus 71 infections in household contacts in Taiwan. J Am Med Assoc.

[3] Zeng M, Khatib NFE, Tu S, Ren P, Xu S, Zhu Q, Mo X, Pu D, Wang X, Altmeyer R (2012). Seroepidemiology of enterovirus 71 infection prior to the 2011 season in children in Shanghai. Journal of Clinical Virology the Official Publication of the Pan American Society for Clinical. Virology.

[4] Lee MS, Chiang PS, Luo ST, Huang ML, Liou GY, Tsao KC, Lin TY (2012). Incidence rates of enterovirus 71 infections in young children during a Nationwide epidemic in Taiwan, 2008–09. PLoS Negl Trop Dis.

[5] Chang LY, Lin TY, Hsu KH, Huang YC, Lin KL, Hsueh C, Shih SR, Ning HC, Hwang MS, Wang HS (1999). Clinical features and risk factors of pulmonary oedema after enterovirus-71-related hand, foot, and mouth disease. Lancet.

[6] The national epidemic situation of notifiable infection disease [http://www.nhc.gov.cn/jkj/index.shtml].

[7] Khetsuriani N, Lamonte-Fowlkes A, Oberst S, Pallansch MA (2006). Enterovirus surveillance--United States, 1970-2005. MMWR Surveill Summ.

[8] Solomon T, Lewthwaite P, Perera D, Cardosa MJ, McMinn P, Ooi MH (2010). Virology, epidemiology, pathogenesis, and control of enterovirus 71. Lancet Infect Dis.

[9] Ooi MH, Wong SC, Lewthwaite P, Cardosa MJ, Solomon T (2010). Clinical features, diagnosis, and management of enterovirus 71. The Lancet Neurology.

[10] Kok CC (2015). Therapeutic and prevention strategies against human enterovirus 71 infection. World journal of virology.

[11] Wu X, Sun Y, Lin C, Jia L, Wu Q, Li X, Wang Q (2014). A case-control study to identify environmental risk factors for hand, foot, and mouth disease outbreaks in Beijing. Jpn J Infect Dis.

[12] Seiff A (2012). Cambodia unravels cause of mystery illness. Lancet.

[13] Tu PV, Thao NT, Perera D, Huu TK, Tien NT, Thuong TC, How OM, Cardosa MJ, McMinn PC (2007). Epidemiologic and virologic investigation of hand, foot, and mouth disease, southern Vietnam, 2005. Emerg Infect Dis.

[14] Ang LW, Koh BK, Chan KP, Chua LT, James L, Goh KT (2009). Epidemiology and control of hand, foot and mouth disease in Singapore, 2001-2007. Ann Acad Med Singap.

[15] Hosoya M, Kawasaki Y, Sato M, Honzumi K, Kato A, Hiroshima T, Ishiko H, Suzuki H (2006). Genetic diversity of enterovirus 71 associated with hand, foot and mouth disease epidemics in Japan from 1983 to 2003. Pediatr Infect Dis J.

[16] Fujimoto T, Chikahira M, Yoshida S, Ebira H, Hasegawa A, Totsuka A, Nishio O (2002). Outbreak of central nervous system disease associated with hand, foot, and mouth disease in Japan during the summer of 2000: detection and molecular epidemiology of enterovirus 71. Microbiol Immunol.

[17] Zhang Y, Tan XJ, Wang HY, Yan DM, Zhu SL, Wang DY, Ji F, Wang XJ, Gao YJ, Chen L (2009). An outbreak of hand, foot, and mouth disease associated with subgenotype C4 of human enterovirus 71 in Shandong, China. Journal of Clinical Virology the Official Publication of the Pan American Society for Clinical. Virology.

[18] Yan Z, Zhen Z, Yang W, Ren J, Tan X, Yu W, Mao N, Xu S, Zhu S, Cui A (2010). An emerging recombinant human enterovirus 71 responsible for the 2008 outbreak of hand foot and mouth disease in Fuyang city of China. Virol J.

[19] Announcement on licensed drugs approved by China Food and Drug Administration. In. China Food and Drug Administration; 2016.

[20] Zheng Y, Jit M, Wu JT, Yang J, Leung K, Liao Q, Yu H (2017). Economic costs and health-related quality of life for hand, foot and mouth disease (HFMD) patients in China. PLoS One.

[21] Wang W, Song J, Wang J, Li Y, Deng H, Li M, Gao N, Zhai S, Dang S, Zhang X (2017). Cost-effectiveness of a national enterovirus 71 vaccination program in China. PLoS Negl Trop Dis.

[22] Zhang Z (2009). Qinghai geography.

[23] Mei D (2013). Qinghai pre-school practical difficulties and countermeasures of the development of the research. Journal of Qinghai normal university(Philosophy and Soc Sci).

[24] Koh WM, Bogich T, Siegel K, Jin J, Chong EY, Tan CY, Chen MI, Horby P, Cook AR (2016). The epidemiology of hand, foot and mouth disease in Asia: a systematic review and analysis. Pediatr Infect Dis J.

[25] Protocol of sample collection and laboratory tests for HFMD cases [http://www.chinacdc.cn/jkzt/crb/szkb/jszl_2275/200906/t20090612_24707.htm].

[26] Health CMo (2009). Guideline for HFMD diagnosis and treatment. edn: China Ministry of Health.

[27] Anselin L (1995). Local indicators of spatial assosiation-LISA. Geogr Anal.

[28] Deng T, Huang Y, Yu S, Gu J, Huang C, Xiao G, Hao Y (2013). Spatial-temporal clusters and risk factors of hand, foot, and mouth disease at the district level in Guangdong Province, China. PLoS One.

[29] Liu L, Zhao X, Yin F, Lv Q (2015). Spatio-temporal clustering of hand, foot and mouth disease at the county level in Sichuan province, China, 2008-2013. Epidemiology & Infection.

[30] Xie YH, Chongsuvivatwong V, Tang Z, Mcneil EB, Tan Y (2014). Spatio-temporal clustering of hand, foot, and mouth disease at the county level in Guangxi, China. PLoS One.

[31] Wang C, Li X, Zhang Y, Xu Q, Huang F, Cao K, Tao L, Guo J, Gao Q, Wang W (2016). Spatiotemporal cluster patterns of hand, foot, and mouth disease at the county level in mainland China, 2008-2012. PLoS One.

[32] Prem K, Cook A, Jit M (2017). Projecting social contact matrices in 152 countries using contact surveys and demographic data. PLoS Comput Biol.

[33] Gui J, Liu Z, Zhang T, Hua Q, Jiang Z, Chen B, Gu H, Lv H, Dong C (2015). Epidemiological characteristics and spatial-temporal clusters of hand, foot, and mouth disease in Zhejiang Province, China, 2008-2012. PLoS One.

[34] Liu Y, Wang X, Liu Y, Sun D, Ding S, Zhang B, Du Z, Xue F (2013). Detecting spatial-temporal clusters of HFMD from 2007 to 2011 in Shandong Province, China. PLoS One.

[35] Shimizu H, Okuyama K, Hirai Y (2005). Epidemic of hand, foot and mouth disease in Kawasaki City, Japan Japanese. Journal of Infectious Diseases.

[36] Podin Y, Gias EL, Ong F, Leong YW, Yee SF, Yusof MA, Perera D, Teo B, Wee TY, Yao SC (2006). Sentinel surveillance for human enterovirus 71 in Sarawak, Malaysia: lessons from the first 7 years. BMC Public Health.

[37] Tang JH, Chan TC, Shigematsu M, Hwang JS (2015). Latitude-based approach for detecting aberrations of hand, foot, and mouth disease epidemics. BMC medical informatics and decision making.

[38] Urashima M, Shindo N, Okabe N (2003). Seasonal models of herpangina and hand-foot-mouth disease to simulate annual fluctuations in urban warming in Tokyo. Jpn J Infect Dis.

[39] Xing W, Liao Q, Viboud C, Zhang J, Sun J, Wu JT, Chang Z, Liu F, Fang VJ, Zheng Y (2014). Hand, foot, and mouth disease in China, 2008–12: an epidemiological study. Lancet Infect Dis.

[40] Dong W, Li X, Yang P, Liao H, Wang X, Wang Q (2016). The effects of weather factors on hand, foot and mouth disease in Beijing. Sci Rep.

[41] Liu Y, Wang X, Pang C, Yuan Z, Li H, Xue F (2015). Spatio-temporal analysis of the relationship between climate and hand, foot, and mouth disease in Shandong province, China, 2008–2012. BMC Infect Dis.

[42] Wang Y, Feng Z, Yang Y, Self S, Gao Y, Longini IM, Wakefield J, Zhang J, Wang L, Chen X (2011). Hand, foot, and mouth disease in China: patterns of spread and transmissibility. Epidemiology.

[43] Bo Y-C, Song C, Wang J-F, Li X-W (2014). Using an autologistic regression model to identify spatial risk factors and spatial risk patterns of hand, foot and mouth disease (HFMD) in mainland China. BMC Public Health.

[44] Takahashi S, Liao Q, Van Boeckel TP, Xing W, Sun J, Hsiao VY, Metcalf CJ, Chang Z, Liu F, Zhang J (2016). Hand, foot, and mouth disease in China: modeling epidemic dynamics of enterovirus serotypes and implications for vaccination. PLoS Med.

[45] Zhao J, Jiang F, Zhong L, Sun J, Ding J (2016). Age patterns and transmission characteristics of hand, foot and mouth disease in China. BMC Infect Dis.

[46] Han JF, Xu S, Zhang Y, Zhu SY, Wu DL, Yang XD, Liu H, Sun BX, Wu XY, Qin CF (2014). Hand, foot, and mouth disease outbreak caused by coxsackievirus A6, China, 2013. J Infect.

[47] Hongyan G, Chengjie M, Qiaozhi Y, Wenhao H, Juan L, Lin P, Yanli X, Hongshan W, Xingwang L (2014). Hand, foot and mouth disease caused by coxsackievirus a6, Beijing, 2013. Pediatr Infect Dis J.

[48] Zhao S, Zhang S, Yue J, Ma R, Jiang S (2011). Analysis of Laboratiorial surveillance on hand-foot-mouth disease in Qinghai Province. Modern Preventive Medicine.

[49] Yang B, Wu P, Wu JT, Lau EH, Leung GM, Yu H, Cowling BJ (2015). Seroprevalence of enterovirus 71 antibody among children in China: a systematic review and meta-analysis. Pediatr Infect Dis J.

[50] Kulldorff M (1997). A spatial scan statistic. Communications in Statistics - Theory and Methods.

[51] Boeckel TPV, Takahashi S, Liao Q, Xing W, Lai S, Hsiao V, Liu F, Zheng Y, Chang Z, Chen Y (2016). Hand, foot, and mouth disease in China: critical community size and spatial vaccination strategies. Sci Rep.

