# Supplementary material for: Epidemiological features and spatial clusters of hand, foot, and mouth disease in Qinghai Province, China, 2009–2015
Source: BMC Infect Dis. 2018 Dec 5;18:624. doi: 10.1186/s12879-018-3509-7 (PMC6280489; doi:10.1186/s12879-018-3509-7)

**Additional File 1**

**Figure** Proportions of enterovirus serotypes in laboratory-confirmed cases of HFMD in 2009-2015, Qinghai Province, China.


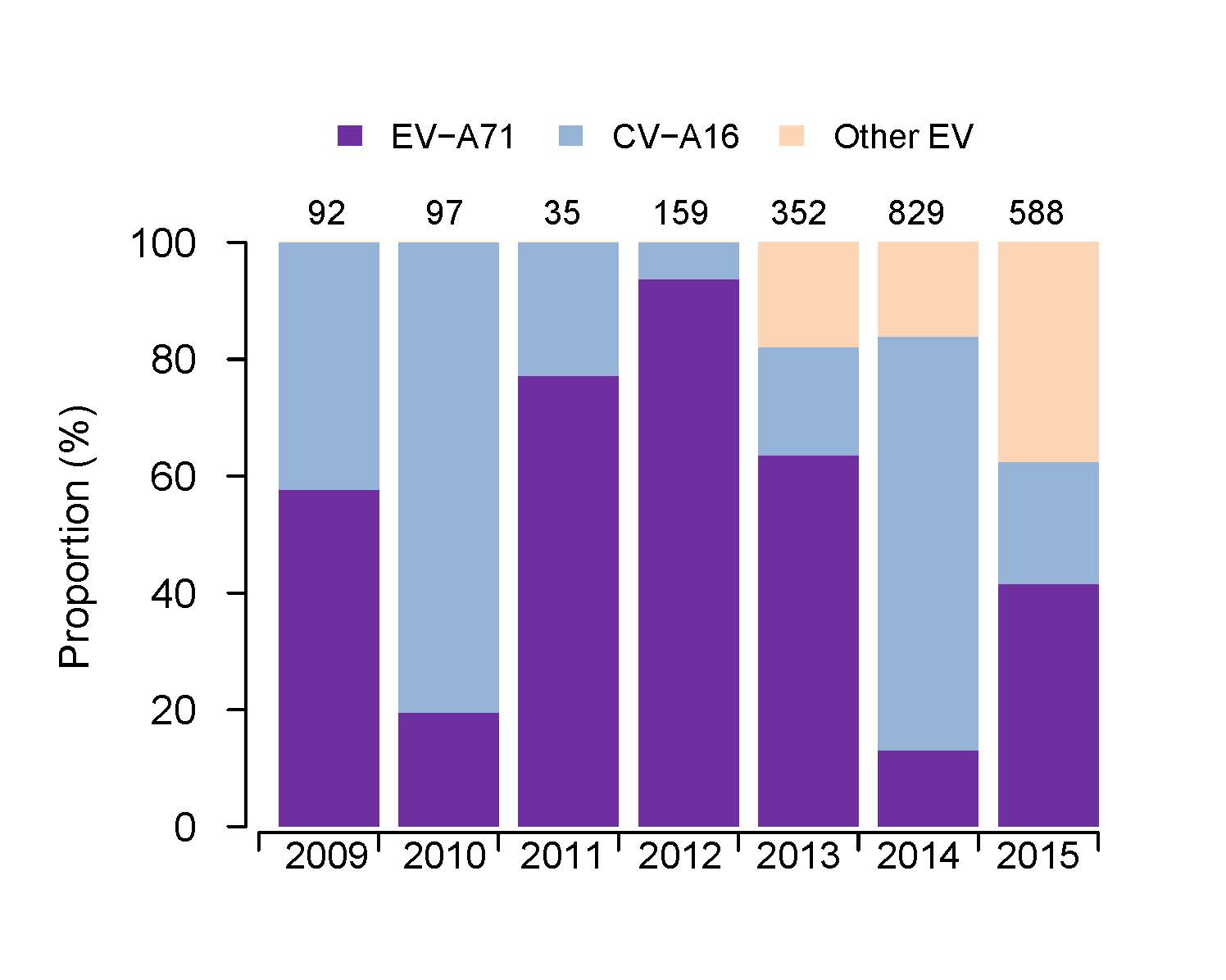

Supplement: Supplementary file 1 — Figure. Proportions of enterovirus serotypes in laboratory-confirmed cases of HFMD in 2009–2015, Qinghai Province, China. (DOCX 96 kb) [file 12879_2018_3509_MOESM1_ESM.docx]
