# Supplementary material for: Epidemiological features and spatial clusters of hand, foot, and mouth disease in Qinghai Province, China, 2009–2015
Source: BMC Infect Dis. 2018 Dec 5;18:624. doi: 10.1186/s12879-018-3509-7 (PMC6280489; doi:10.1186/s12879-018-3509-7)
Supplement: Supplementary file 2 — Table. Annual incidence rates, severity, and case-fatality for children 0–5 years of age of Hand, Foot, and Mouth Disease (HFMD), Qinghai Province, 2009–2015. (DOCX 13 kb) [file 12879_2018_3509_MOESM2_ESM.docx]

**Additional File 2**

**Table** Annual incidence rates, severity, and case-fatality for children 0-5 years of age of Hand, Foot, and Mouth Disease (HFMD), Qinghai Province, 2009–2015.

| **Year** | **Number of Cases** | **Number of Severe Cases** | **Number of Deaths** | **Incidence Rate（/100,000）** | **Percent of cases with severe disease（%）** | **Case-fatality（%）*** |
| --- | --- | --- | --- | --- | --- | --- |
| 2009 | 2,436 | 0 | 0 | 44.0 | 0.00 | 0.00 |
| 2010 | 4,561 | 1 | 0 | 81.8 | 0.02 | 0.00 |
| 2011 | 603 | 0 | 0 | 10.7 | 0.00 | 0.00 |
| 2012 | 792 | 0 | 0 | 13.9 | 0.00 | 0.00 |
| 2013 | 1,681 | 1 | 1 | 29.4 | 0.06 | 0.06 |
| 2014 | 2,680 | 3 | 1 | 46.4 | 0.11 | 0.04 |
| 2015 | 1,727 | 2 | 1 | 29.6 | 0.12 | 0.06 |
| **Total** | **14,480** | **7** | **3** | **36.4** | **0.05** | **0.02** |
