# Supplementary material for: Epidemiological features and spatial clusters of hand, foot, and mouth disease in Qinghai Province, China, 2009–2015
Source: BMC Infect Dis. 2018 Dec 5;18:624. doi: 10.1186/s12879-018-3509-7 (PMC6280489; doi:10.1186/s12879-018-3509-7)
Supplement: Supplementary file 3 — Figure. Annual and mean seven-year county level incidence rates of HFMD, Qinghai Province, China, 2009–2015. (DOCX 220 kb) [file 12879_2018_3509_MOESM3_ESM.docx]

**Additional File 3**

**Figure** Annual and mean seven-year county level incidence rates of HFMD, Qinghai Province, China, 2009-2015.

**
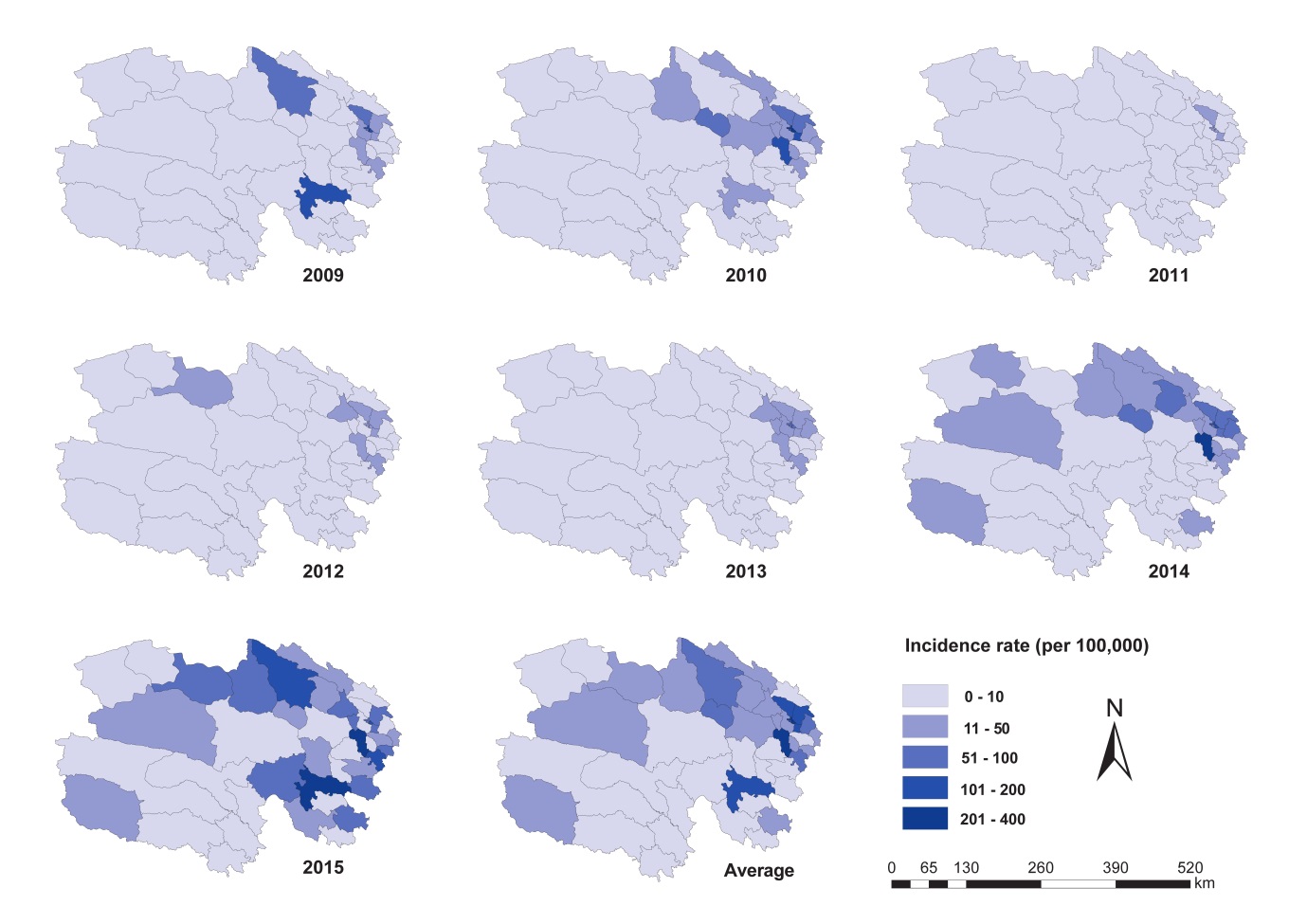
**
