# Supplementary material for: Epidemiological features and spatial clusters of hand, foot, and mouth disease in Qinghai Province, China, 2009–2015
Source: BMC Infect Dis. 2018 Dec 5;18:624. doi: 10.1186/s12879-018-3509-7 (PMC6280489; doi:10.1186/s12879-018-3509-7)
Supplement: Supplementary file 4 — Tables S1–7. Statistically significant county-level high incidence and low incidence spatial clusters of Hand, Foot, and Mouth Disease identified using the Local Indicator for Spatial Autocorrelation in GeoDa software in 2009–2015. (DOCX 24 kb) [file 12879_2018_3509_MOESM4_ESM.docx]

**Additional File 4**

**Table 1** Statistically significant county-level high incidence and low incidence spatial clusters of Hand, Foot, and Mouth Disease identified using the Local Indicator for Spatial Autocorrelation in GeoDa software in 2009.

| Type | County Name | Incidence Rate | Local Moran | *Ｐ-*value |
| --- | --- | --- | --- | --- |
| High-Risk Cluster | Chengdong | 2110.3 | 3.06 | 0.002 |
|  | Huangzhong | 363.9 | 0.01 | 0.001 |
|  | Chengzhong | 5057.3 | 6.97 | 0.001 |
|  | Chengbei | 2503.5 | 3.52 | 0.001 |
|  | Chengxi | 1211.4 | 2.43 | 0.002 |
| Low-Risk Cluster | Chindu | 0.0 | 0.16 | 0.026 |
|  | Qumarleeb | 0.0 | 0.15 | 0.027 |
|  | Yushu | 0.0 | 0.16 | 0.001 |
|  | Golmud | 88.3 | 0.12 | 0.001 |

**Table 2** Statistically significant county-level high incidence and low incidence spatial clusters of Hand, Foot, and Mouth Disease identified using the Local Indicator for Spatial Autocorrelation in GeoDa software in 2010.

| Type | County Name | Incidence Rate | Local Moran | *Ｐ-*value |
| --- | --- | --- | --- | --- |
| High-Risk Cluster | Huzhu | 778.1 | 0.10 | 0.006 |
|  | Chengdong | 5271.2 | 4.86 | 0.001 |
|  | Chengzhong | 7056.5 | 7.92 | 0.001 |
|  | Chengbei | 5631.0 | 4.69 | 0.001 |
|  | Chengxi | 2971.1 | 3.65 | 0.001 |
| Low-Risk Cluster | Maqeen | 198.9 | 0.12 | 0.020 |
|  | Chindu | 0.0 | 0.18 | 0.018 |
|  | Zhidoi | 37.7 | 0.16 | 0.026 |
|  | Nangqeen | 0.0 | 0.18 | 0.045 |
|  | Qumarleeb | 0.0 | 0.17 | 0.022 |
|  | Yushu | 0.0 | 0.17 | 0.008 |
|  | Madoi | 0.0 | 0.16 | 0.035 |
|  | Golmud | 90.7 | 0.15 | 0.009 |

**Table 3** Statistically significant county-level high incidence and low incidence spatial clusters of Hand, Foot, and Mouth Disease identified using the Local Indicator for Spatial Autocorrelation in GeoDa software in 2011.

| Type | County Name | Incidence Rate | Local Moran | *Ｐ-*value |
| --- | --- | --- | --- | --- |
| High-Risk Cluster | Chengdong | 684.6 | 2.69 | 0.001 |
|  | Chengzhong | 1619.7 | 7.23 | 0.001 |
|  | Chengbei | 697.2 | 2.88 | 0.001 |
|  | Chengxi | 648.9 | 4.29 | 0.004 |
| Low-Risk Cluster | Xinghai | 0.0 | 0.15 | 0.018 |
|  | Maqeen | 19.9 | 0.13 | 0.005 |
|  | Qumarleeb | 0.0 | 0.16 | 0.009 |
|  | Yushu | 0.0 | 0.16 | 0.009 |
|  | Madoi | 0.0 | 0.16 | 0.021 |
|  | Zadoi | 0.0 | 0.16 | 0.049 |

**Table 4** Statistically significant county-level high incidence and low incidence spatial clusters of Hand, Foot, and Mouth Disease identified using the Local Indicator for Spatial Autocorrelation in GeoDa software in 2012.

| Type | County Name | Incidence Rate | Local Moran | *Ｐ-*value |
| --- | --- | --- | --- | --- |
| High-Risk Cluster | Ping'an | 210.9 | 0.66 | 0.009 |
|  | Huzhu | 574.1 | 2.46 | 0.007 |
|  | Chengdong | 762.0 | 4.57 | 0.001 |
|  | Datong | 240.0 | 0.61 | 0.040 |
|  | Chengzhong | 447.1 | 3.20 | 0.002 |
|  | Chengbei | 325.5 | 2.22 | 0.001 |
| Low-Risk Cluster | Maqeen | 60.2 | 0.14 | 0.036 |
|  | Qumarleeb | 0.0 | 0.32 | 0.023 |
|  | Yushu | 13.3 | 0.31 | 0.015 |
|  | Madoi | 0.0 | 0.32 | 0.037 |

**Table 5** Statistically significant county-level high incidence and low incidence spatial clusters of Hand, Foot, and Mouth Disease identified using the Local Indicator for Spatial Autocorrelation in GeoDa software in 2013.

| Type | County Name | Incidence Rate | Local Moran | *Ｐ-*value |
| --- | --- | --- | --- | --- |
| High-Risk Cluster | Ping'an | 533.0 | 0.55 | 0.028 |
|  | Huzhu | 610.0 | 0.98 | 0.001 |
|  | Chengdong | 1560.3 | 4.76 | 0.001 |
|  | Datong | 388.6 | 0.26 | 0.031 |
|  | Huangzhong | 372.8 | 0.45 | 0.001 |
|  | Chengzhong | 1459.9 | 6.81 | 0.001 |
|  | Chengbei | 1492.7 | 5.25 | 0.001 |
|  | Chengxi | 1617.2 | 6.55 | 0.003 |
| Low-Risk Cluster | Tongde | 31.7 | 0.20 | 0.048 |
|  | Maqeen | 42.2 | 0.20 | 0.007 |
|  | Jigzhi | 39.3 | 0.21 | 0.017 |
|  | Chindu | 0.0 | 0.26 | 0.017 |
|  | Qumarleeb | 0.0 | 0.24 | 0.022 |
|  | Yushu | 0.0 | 0.25 | 0.003 |
|  | Madoi | 0.0 | 0.24 | 0.019 |
|  | Dulan | 17.2 | 0.20 | 0.036 |
|  | Golmud | 82.7 | 0.15 | 0.024 |

**Table 6** Statistically significant county-level high incidence and low incidence spatial clusters of Hand, Foot, and Mouth Disease identified using the Local Indicator for Spatial Autocorrelation in GeoDa software in 2014.

| Incidence Rate | County Name | Incidence Rate | Local Moran | *Ｐ-*value |
| --- | --- | --- | --- | --- |
| High-Risk Cluster | Huzhu | 602.2 | 0.39 | 0.002 |
|  | Chengdong | 2111.1 | 2.85 | 0.009 |
|  | Chengzhong | 1324.3 | 2.66 | 0.002 |
|  | Chengbei | 2076.2 | 3.07 | 0.003 |
|  | Chengxi | 1553.1 | 2.41 | 0.027 |
| Low-Risk Cluster | Tongde | 15.4 | 0.40 | 0.030 |
|  | Xinghai | 11.8 | 0.38 | 0.026 |
|  | Maqeen | 42.8 | 0.37 | 0.010 |
|  | Nangqeen | 0.0 | 0.44 | 0.031 |
|  | Yushu | 6.7 | 0.43 | 0.002 |
|  | Madoi | 88.0 | 0.33 | 0.008 |
|  | Golmud | 402.3 | 0.00 | 0.002 |
|  | Lenghu | 0.0 | 0.45 | 0.032 |

**Table 7** Statistically significant county-level high incidence and low incidence spatial clusters of Hand, Foot, and Mouth Disease identified using the Local Indicator for Spatial Autocorrelation in GeoDa software in 2015.

| Type | County Name | Incidence Rate | Local Moran | *Ｐ-*value |
| --- | --- | --- | --- | --- |
| High-Risk Cluster | Chengzhong | 750.6 | 1.54 | 0.008 |
|  | Chengbei | 647.8 | 0.85 | 0.010 |
| Low-Risk Cluster | Zhidoi | 55.9 | 0.57 | 0.046 |
|  | Nangqeen | 76.7 | 0.81 | 0.002 |
|  | Qumarleeb | 116.7 | 0.42 | 0.041 |
|  | Yushu | 6.6 | 0.89 | 0.002 |
